# Supplementary material for: An integrative machine learning approach to discovering multi-level molecular mechanisms of obesity using data from monozygotic twin pairs
Source: R Soc Open Sci. 2020 Oct 21;7(10):200872. doi: 10.1098/rsos.200872 (PMC7657920; doi:10.1098/rsos.200872)
Supplement: Figure S1 [file rsos200872supp1.pdf]

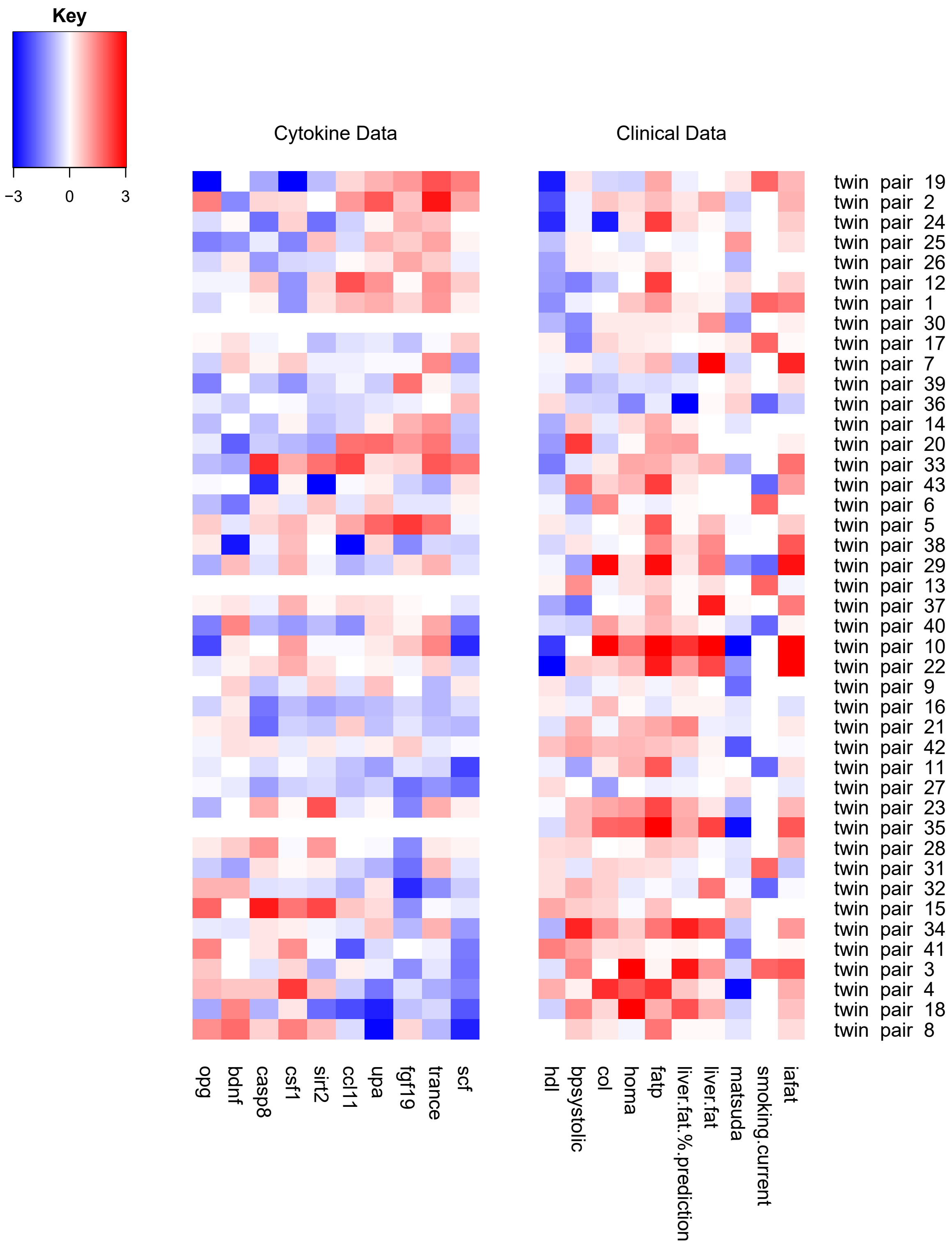

Key
−3
0
3
Cytokine Data
Clinical Data
twin pair 19
twin pair 2
twin pair 24
twin pair 25
twin pair 26
twin pair 12
twin pair 1
twin pair 30
twin pair 17
twin pair 7
twin pair 39
twin pair 36
twin pair 14
twin pair 20
twin pair 33
twin pair 43
twin pair 6
twin pair 5
twin pair 38
twin pair 29
twin pair 13
twin pair 37
twin pair 40
twin pair 10
twin pair 22
twin pair 9
twin pair 16
twin pair 21
twin pair 42
twin pair 11
twin pair 27
twin pair 23
twin pair 35
twin pair 28
twin pair 31
twin pair 32
twin pair 15
twin pair 34
twin pair 41
twin pair 3
twin pair 4
twin pair 18
twin pair 8
opg
bdnf
casp8
csf1
sirt2
ccl11
upa
fgf19
trance
scf
hdl
bpsystolic
col
homa
fatp
liver.fat.%.prediction
liver.fat
matsuda
smoking.current
iafat
