# Supplementary material for: An integrative machine learning approach to discovering multi-level molecular mechanisms of obesity using data from monozygotic twin pairs
Source: R Soc Open Sci. 2020 Oct 21;7(10):200872. doi: 10.1098/rsos.200872 (PMC7657920; doi:10.1098/rsos.200872)
Supplement: Figure S2 [file rsos200872supp2.pdf]

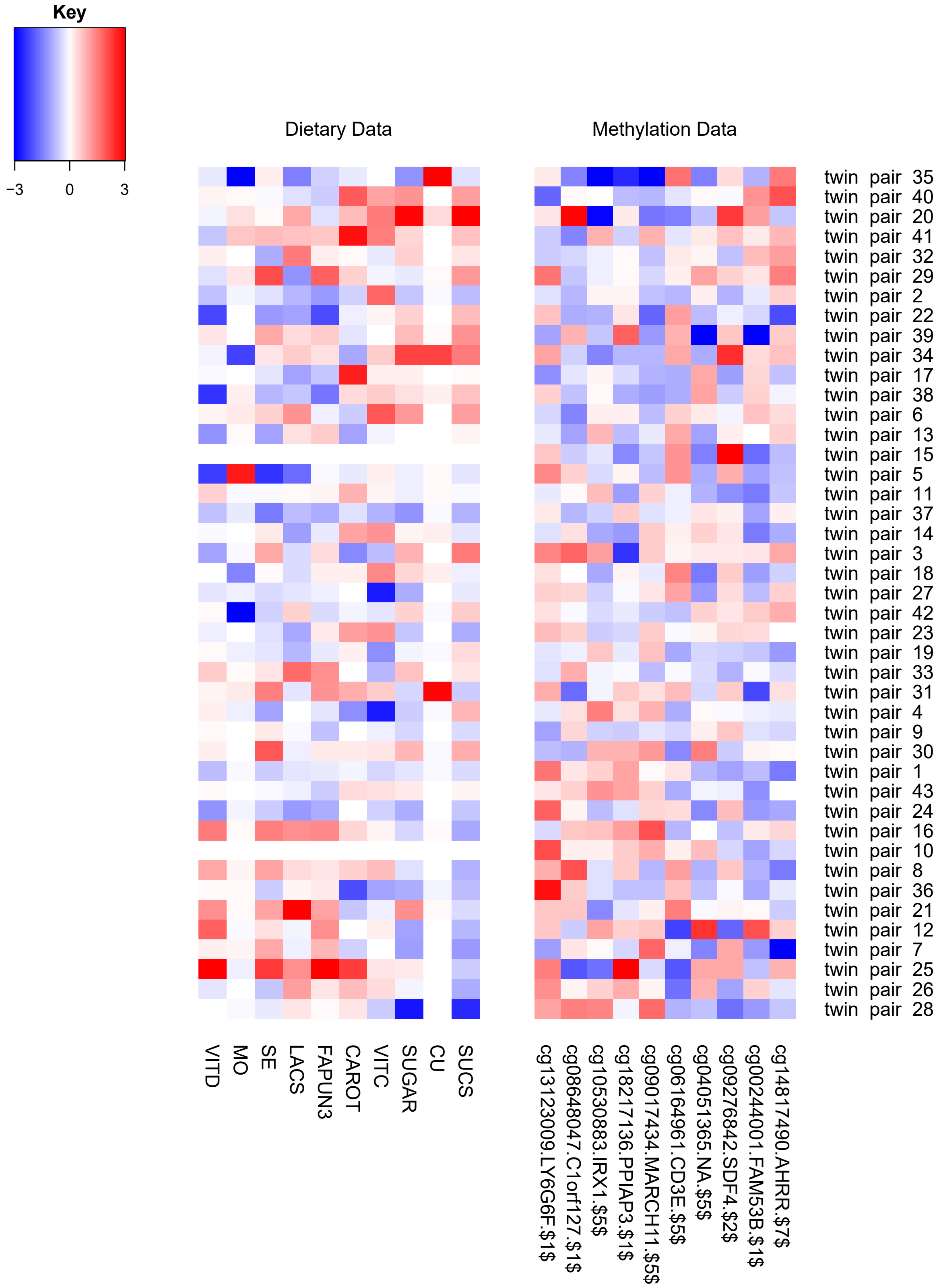

Key
−3
0
3
Dietary Data
Methylation Data
twin pair 35
twin pair 40
twin pair 20
twin pair 41
twin pair 32
twin pair 29
twin pair 2
twin pair 22
twin pair 39
twin pair 34
twin pair 17
twin pair 38
twin pair 6
twin pair 13
twin pair 15
twin pair 5
twin pair 11
twin pair 37
twin pair 14
twin pair 3
twin pair 18
twin pair 27
twin pair 42
twin pair 23
twin pair 19
twin pair 33
twin pair 31
twin pair 4
twin pair 9
twin pair 30
twin pair 1
twin pair 43
twin pair 24
twin pair 16
twin pair 10
twin pair 8
twin pair 36
twin pair 21
twin pair 12
twin pair 7
twin pair 25
twin pair 26
twin pair 28
VITD
MO
SE
LACS
FAPUN3
CAROT
VITC
SUGAR
CU
SUCS
cg13123009.LY6G6F.1
cg08648047.C1orf127.1
cg10530883.IRX1.5
cg18217136.PPIAP3.1
cg09017434.MARCH11.5
cg06164961.CD3E.5
cg04051365.NA.5
cg09276842.SDF4.2
cg00244001.FAM53B.1
cg14817490.AHRR.7
