# Supplementary material for: An integrative machine learning approach to discovering multi-level molecular mechanisms of obesity using data from monozygotic twin pairs
Source: R Soc Open Sci. 2020 Oct 21;7(10):200872. doi: 10.1098/rsos.200872 (PMC7657920; doi:10.1098/rsos.200872)
Supplement: Figure S3 [file rsos200872supp3.pdf]

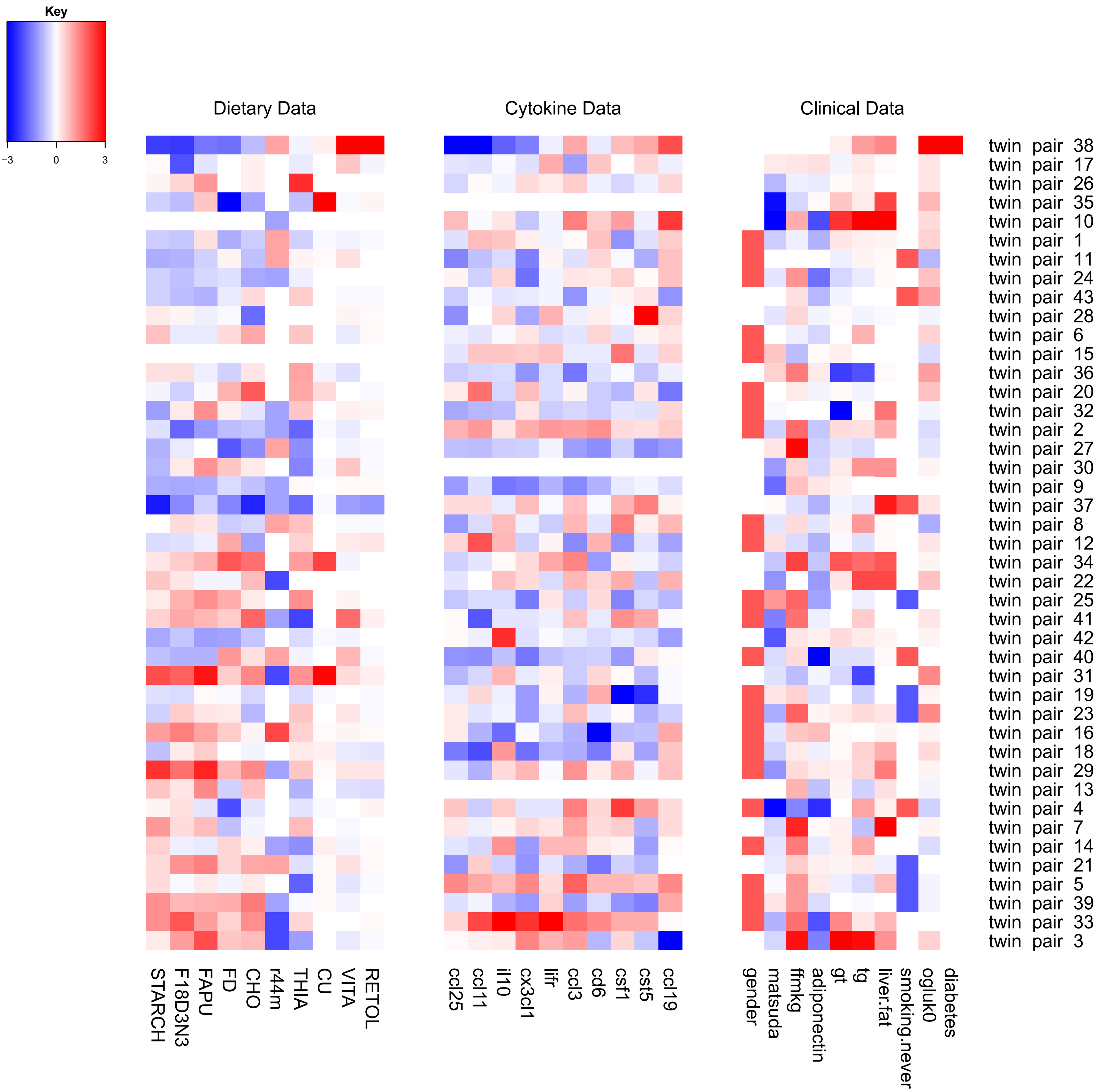

Key
−3
0
3
Dietary Data
Cytokine Data
Clinical Data
STARCH
F18D3N3
FAPU
FD
CHO
r44m
THIA
CU
VITA
RETOL
ccl25
ccl11
il10
cx3cl1
lifr
ccl3
cd6
csf1
cst5
ccl19
gender
matsuda
ffmkg
adiponectin
gt
tg
liver.fat
smoking.never
ogluk0
diabetes
twin pair 38
twin pair 17
twin pair 26
twin pair 35
twin pair 10
twin pair 1
twin pair 11
twin pair 24
twin pair 43
twin pair 28
twin pair 6
twin pair 15
twin pair 36
twin pair 20
twin pair 32
twin pair 2
twin pair 27
twin pair 30
twin pair 9
twin pair 37
twin pair 8
twin pair 12
twin pair 34
twin pair 22
twin pair 25
twin pair 41
twin pair 42
twin pair 40
twin pair 31
twin pair 19
twin pair 23
twin pair 16
twin pair 18
twin pair 29
twin pair 13
twin pair 4
twin pair 7
twin pair 14
twin pair 21
twin pair 5
twin pair 39
twin pair 33
twin pair 3
