# Supplementary figures and images for: An integrative machine learning approach to discovering multi-level molecular mechanisms of obesity using data from monozygotic twin pairs
Source: R Soc Open Sci. 2020 Oct 21;7(10):200872. doi: 10.1098/rsos.200872 (PMC7657920; doi:10.1098/rsos.200872)

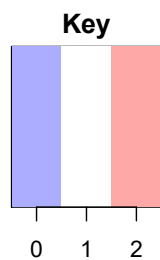

Genotype Data

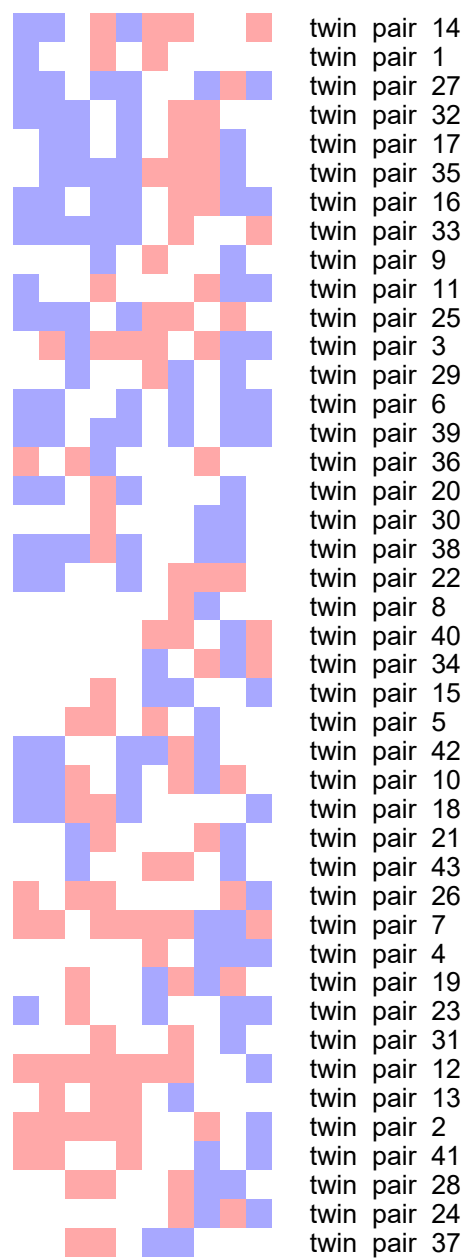

rs11126185 G LOC391383\_4  
rs644587 T IL12A\_4  
rs972283 G KLF14\_3  
rs10742752 C SYT13\_2  
rs1337332 C HS3ST5\_2  
rs1421085 C FTO\_2  
rs552976 G G6PC2,ABCB11\_3  
rs13083798 A PRBM1\_2  
rs1558902 A FTO\_1  
rs11642841 A FTO\_3

Supplement: Figure S5 [file rsos200872supp5.pdf]
